# Patterns and predictors of adaptive skills in 2- to 7-year-old children with Down syndrome

**DOI:** 10.1186/s11689-022-09430-4

**Published:** 2022-03-12

**Authors:** Emily K. Schworer, Anna J. Esbensen, Vivian Nguyen, Lauren Bullard, Deborah J. Fidler, Lisa A. Daunhauer, Carolyn B. Mervis, Angela M. Becerra, Leonard Abbeduto, Angela John Thurman

**Affiliations:** 1 Division of Developmental and Behavioral Pediatrics, Cincinnati Children’s Hospital Medical Center, Cincinnati, OH USA; 2 Department of Pediatrics, University of Cincinnati College of Medicine, Cincinnati, OH USA; 3 University of California Davis Health, MIND Institute and Department of Psychiatry and Behavioral Sciences, Sacramento, CA USA; 4 Human Development and Family Studies, Colorado State University, Fort Collins, CO USA; 5 Department of Psychological and Brain Sciences, University of Louisville, Louisville, KY USA

**Keywords:** Down syndrome, Adaptive skills, Motor skills, Cognition, ASD symptoms

## Abstract

**Background:**

There is substantial variability in adaptive skills among individuals with Down syndrome. Few studies, however, have focused on the early developmental period or on the potential sources of variability in adaptive skills. This study characterizes adaptive skills in young children with Down syndrome and investigates child characteristics associated with adaptive skills.

**Methods:**

Participants were 44 children with Down syndrome ranging in age from 2.50 to 7.99 years (*M* = 4.66 years, *SD* = 1.46). The Vineland Adaptive Behavior Scales-3 (VABS-3) Comprehensive Interview Form was used to assess adaptive behavior in the three core domains: socialization, daily living, and communication skills. Caregivers also reported on motor skills and autism spectrum disorder symptoms. Child cognitive abilities were assessed.

**Results:**

Analyses comparing mean standard score performance across the three VABS-3 core domains demonstrated significant differences between all pairs of domains, resulting in a group-level pattern of socialization > daily living > communication skills. At the individual level, 10 different patterns of relative strength and weakness were identified, with only 18% of participants evidencing significant differences between adaptive skill domain standard scores corresponding to the group-level pattern of significant differences. Child characteristics (cognitive abilities, motor skills, and autism spectrum disorder symptoms) were significantly associated with VABS-3 adaptive domain standard scores.

**Conclusion:**

These findings underscore the importance of individualizing intervention programs focused on improving the adaptive skills of young children with Down syndrome based on consideration of the child’s relative adaptive strengths and weaknesses.

## Background

With a prevalence of 1 in 707 live births, Down syndrome (DS) is the most common neurogenetic syndrome associated with intellectual disability [26]. Individuals with DS demonstrate a great deal of phenotypic heterogeneity at all developmental stages [22]. At the same time, common areas of challenge are often observed, including delays in nonverbal cognitive, verbal, and motor skill development [8] and increased rates of autism spectrum disorder (ASD) symptoms [5, 13]. Each of these areas can impact adaptive skills (i.e., the conceptual, social, and practical skills people learn and use in their daily lives). The development of adaptive skills is delayed in most individuals with DS, and challenges with these skills can limit an individual’s ability to function independently [42, 46]. Elucidating the nature of adaptive skills in young children with DS and the factors supporting their development will provide the foundation for targeted early interventions to enhance and support later independence.

### Patterns of adaptive skills in children with Down syndrome

Adaptive skills have been examined in DS using caregiver report measures focused on the communication, daily living, and socialization domains. Although delays are typically observed in all areas of adaptive behavior, findings suggest that patterns of relative strength and challenge may emerge when comparing adaptive skills across domains. For example, socialization skills, defined as play, interpersonal, and coping skills, are often reported to be an area of relative strength when compared to communication skills [11, 16, 19, 21, 28]. Moreover, this pattern appears to emerge by the toddler years [19, 46].

More variable findings have been reported when comparing daily living skills, defined as self-care activities and routines [38], to either socialization or communication skills. Daily living skill abilities commonly fall between socialization abilities and communication abilities, but these differences are not always statistically significant [16, 19, 21, 28]. With regard to those studies considering daily living skills in young children with DS, both Fidler et al. [19], who compared domain-level age-equivalent scores, and Will et al. [46], who compared standard scores, reported daily living skill scores that fell below scores in the socialization domain but above scores in the communication domain. Only Fidler et al. [19] statistically compared adaptive skills across domains, finding that daily living age-equivalent scores were not significantly different from either socialization age-equivalent scores or communication age-equivalent scores.

Although findings from these studies have shed light on the patterns observed across adaptive skill domains for children with DS, there are still significant gaps in our understanding of the nature of these patterns. For example, many studies conducted to date have included broad age ranges in their participant samples [12, 16, 35], thereby masking possible differences in patterns as a function of developmental period. In addition, much of the research conducted to date has utilized age-equivalent scores to deal with floor effects in standard score performance. Unfortunately, the psychometric properties of age-equivalent scores preclude comparisons between different domains of functioning or children of different chronological ages because these scores are not on an equal-interval scale. In particular, similar changes in raw scores often result in very different changes in age-equivalent scores as a function of domain, and the amount of change in age-equivalent as a function of a 1-point increase in raw score is not consistent even within a single domain (e.g., [17, 29–31, 36]). Finally, although extant research highlights considerable heterogeneity of adaptive skills among children with DS [42, 43], no studies to date have examined the extent to which the group-level patterns of performance across adaptive skill domains are representative of performance at the level of the individual with DS. In the present study, we sought to address these gaps in the literature by characterizing patterns of performance using standard scores, which do not suffer from the psychometric limitations associated with age-equivalent scores, and describing patterns of relative strength and challenge at the individual level to identify subgroups of children who may be overlooked when utilizing an aggregated method only.

### Common areas of challenge in Down syndrome

In addition to characterizing the patterns of relative strength and weakness across adaptive skill domains, it is also important to identify sources of within-group variability. Although our current understanding of the factors contributing to the development of adaptive skills in young children with DS remains limited, several factors have been implicated as potential key contributors.

#### Cognitive ability

Because the overall degree of cognitive impairment varies among individuals with DS, individual differences in cognition are often considered when investigating developmental trajectories and adaptive skill outcomes. Marchal et al. [28] used a measure of nonverbal cognition as a predictor of variability in adaptive skills examined longitudinally in DS and found that higher nonverbal mental age at a chronological age of 2 years predicted better adaptive skills at a chronological age of 10 years. Cross-sectional studies also show an association between cognitive ability and adaptive skills in children with DS [24, 34]. Despite these findings, there remains much to learn about the relation between cognition and adaptive skills in young children with DS, including the possibility that this association might vary as a function of other child characteristics such as motor skills and symptoms of ASD.

#### Motor skills

Motor skills develop in the same order albeit at a slower pace in children with DS compared to children with typical development [33, 47, 48]. There is also a wider age range in which particular motor milestones are achieved in DS relative to the general population [48]. These differences in motor development may be relevant to adaptive skill outcomes, as previous work with both children with typical development and children with ASD has identified links between both fine and gross motor skills and adaptive skills, school achievement, and language outcomes [2, 3, 23, 25]. Although the relation between motor and adaptive skills has not been investigated in DS, several studies have observed a relation between motor skills and overall developmental skills or functional status in young children with DS [27, 43]. It remains to be determined whether a similar relation exists between motor skills and domains of adaptive skills, particularly during early development.

#### Autism spectrum disorder symptoms

Although social relatedness is an area of relative strength for many children with DS [20] and socialization is often reported to be a relative adaptive strength in this population, these relative strengths do not preclude social difficulties [4, 5]. Individuals with DS demonstrate challenges navigating social interactions, and individuals with DS are at greater risk for presenting with symptoms of ASD relative to the general population [5, 7, 13]. Indeed, children with DS show a greater incidence of co-occurring ASD than children in the general population (18% versus ~1%) [13]. Moreover, relative to individuals with typical development, increased rates of ASD symptoms are observed even in individuals with DS who do not have a co-occurring ASD diagnosis [5]. Finally, ASD symptomology is negatively associated with adaptive skills in studies examining co-occurring ASD in DS. More specifically, children and adults with co-occurring ASD and DS have been shown to have lower communication, daily living, and socialization adaptive skills compared to individuals with DS without ASD [15, 32]. Because children with DS, even when not formally diagnosed with ASD, show more ASD symptoms than children with typical development [5], the current study focuses on the association between ASD symptomatology as a continuous measure and adaptive skills to provide insight into this relation in young children with DS more broadly.

### Present study

In the present study, we evaluated adaptive skills using the Vineland Adaptive Behavior Scales-3 Comprehensive Interview Form. The study was designed to characterize patterns across adaptive skill domains in young children with DS ages 2.5–7.99 years old at both the group level and the individual child level. We also considered other common areas of challenge in DS hypothesized to be associated with adaptive skills: cognitive ability, motor skills, and ASD symptomatology and assessed their unique contributions to adaptive skill performance. Specifying which child characteristics contribute unique variance to adaptive skills will inform our understanding of the nature of adaptive skills in young children with DS and identify factors that may support the development of independence across different domains and thus, potential pathways for treatment. Specifically, we considered two research questions:

1. Are there significant differences in performance across the VABS-3 adaptive skill domains? We first considered the group-level pattern of relative strengths and weaknesses across adaptive skill domains and then conducted follow-up analyses to consider the extent to which performance at the individual level reflected group-level patterns. We hypothesized that a significant effect of adaptive skill domain would be observed at the group level and that follow-up analyses considering patterns observed at the individual level would reveal significantly more variability than is suggested by the group-level findings.
2. Are individual differences in socialization, daily living, and communication adaptive skills predicted by the simultaneous consideration of concurrent child characteristics (chronological age, cognitive ability, motor skills, and ASD symptomatology)? We hypothesized that a significant amount of variance in adaptive skill domains would be accounted for by this combination of predictors.

## Method

### Study design and procedures

The Institutional Review Boards at each site in this multisite study approved all procedures. Participants were recruited through mailings, social media, and flyer distributions at local DS organizations. To be eligible for the study, participants were required to be 2.5–7.99 years old, have genetic confirmation of a DS diagnosis, use English as the primary language in the home, and have no uncorrected visual or hearing impairments. Participants also were required to have basic motor competencies, including the ability to transition in and out of sitting positions independently, reach for toys, and be independently mobile (i.e., able to scoot, crawl, or walk) to support completion of study procedures. Caregivers provided a karyotype or other medical documentation of DS to confirm the diagnosis. Data from the current study were drawn from the first visit of a larger longitudinal study. During the visit, children’s cognitive skills were assessed. Caregivers also reported on child adaptive skills, motor skills, and ASD symptomatology.

### Participants

Participants were 44 children (22 females) with DS ranging in age from 2.57 to 7.98 years (*M* = 4.66 years, *SD* = 1.46). Participants primarily had Trisomy 21/nondisjunction (90.9%), but there were also participants with mosaic (2.3%) and translocation (4.5%) forms of DS. The type of DS was unknown for one child (2.3%) due to incomplete medical records. Participant demographics are presented in Table 1.

**Table 1:** Participant demographics (*n* = 44)

	***n*** (%)
Race and Ethnicity	
White and non-Hispanic	31 (70.5%)
White and Hispanic	5 (11.3%)
Asian and non-Hispanic	2 (4.5%)
Asian and Hispanic	1 (2.3%)
Other and Hispanic	2 (4.5%)
Multiracial and Hispanic	1 (2.3%)
White and no reported ethnicity	1 (2.3%)
Preferred not to answer	1 (2.3%)
Annual family income (USD)	
< $50,000	3 (6.8%)
$50,001–100,000	11 (25.0%)
$100,001–150,000	15 (34.1%)
> $150,000	12 (27.2%)
Preferred not to answer	3 (6.8%)

### Measures

#### Vineland adaptive behavior scales, third edition comprehensive interview form (VABS-3 [36];)

Adaptive skills were assessed using the VABS-3 semi-structured caregiver interview. Caregiver responses indicate the degree to which participants completed each item without assistance (beyond what may be described for an item) or prompts on an ordinal scale: 2 (often), 1 (sometimes), or 0 (never). The measure assesses three core domains of adaptive skills: communication, daily living skills, and socialization. The communication domain is made up of the receptive, expressive, and written language subdomains. The daily living skills domain consists of personal, domestic, and community adaptive skills. The socialization domain includes interpersonal relationships, play and leisure, and coping skills. The VABS-3 calculates an overall Adaptive Behavior Composite (ABC) comprised of performance on the communication, daily living skills, and socialization domains. The optional Motor Skills domain consists of fine and gross motor subdomains. For the general population, mean domain standard scores on the VABS-3 are 100 (*SD* =15). In the VABS-3 manual, standard scores are classified into low (20–70), moderately low (71–85), adequate (86–114), moderately high (115–129), and high (130–140) categories. The caregiver interview demonstrates high test-retest reliability (*r =* 0.76 – 0.89) and internal consistency (*α* = 0.90–0.98) and the VABS-3 has been deemed appropriate for use in DS by expert consensus [18]. Data from the VABS-3 were used to assess communication, daily living skills, and socialization skills using standard scores. VABS-3 Pairwise Difference Comparisons, one of the scoring outputs provided by the VABS-3 scoring software, were also completed for each participant to assess whether there were significant differences (*p* < .05) between pairs of domain standard scores. Motor skills were also assessed using the VABS-3 and standard scores from this domain were used as an independent variable in subsequent analyses. The motor skills domain was not administered to caregivers of two children due to examiner error.

#### Differential ability scales, second edition (DAS-II [17];)

The DAS-II is a standardized measure of verbal and nonverbal intelligence for children ages 2.5–17.99 years old. All participants completed the Early Years forms, which provide an estimate of General Conceptual Ability (GCA). For children aged 2.5–3.49 years, four subtests contribute to the GCA: verbal comprehension, picture similarities, naming vocabulary, and pattern construction. For children 3.5 years of age and older, two additional subtests: copying and matrices also contribute to the GCA. For the general population, the mean GCA standard score is 100 (*SD* =15). The DAS-II GCA demonstrates adequate internal consistency (*α* = 0.93–0.96) and has been used in previous studies involving children with DS [1, 41].

#### Social responsiveness scale, second edition (SRS-2 [10];)

The SRS-2 includes 65 items related to social awareness, social cognition, social communication, social motivation, and restricted interests and repetitive behavior to assess the severity of overall social deficits through caregiver report. Caregivers respond using an ordinal scale that ranges from 1 (not true) to 4 (almost always true). The SRS-2 has been validated with other autism measurement tools (i.e., Autism Diagnostic Observation Schedule, Autism Diagnostic Interview-Revised, and Social Communication Questionnaire) [7, 9] and demonstrates high internal consistency (*α* = 0.94–0.96) [10]. In accordance with the SRS-2 manual, the preschool form was used for children ages 2.5–4.49 years old and the school-age form for children ages 4.5 years and older. The SRS-2 total *T*-score (*M* = 50, *SD* = 10) was used to estimate ASD symptomatology. Higher scores indicate greater ASD symptomatology.

### Analysis plan

Our first aim was to characterize performance across adaptive skill domains in young children with DS. First, at the group level, mean standard scores for the VABS-3 communication, daily living skills, and socialization domains were compared using a repeated-measures ANOVA to determine if significant differences could be detected across domains. In the presence of a main effect, follow-up post hoc analyses, using a Bonferroni correction, were conducted to clarify the presence of differences between standard scores for each pair of domains. In addition, at the individual level, VABS-3 Pairwise Difference Comparisons were evaluated and used to characterize the relations between standard scores on each of the three core adaptive skill domains for each participant.

Our second aim was to address the contribution of concurrent participant characteristics (chronological age, cognitive ability, motor skills, and ASD symptomatology) to individual differences in VABS-3 communication, daily living skills, and socialization standard scores. First, we used bivariate Pearson correlations to consider the relations between each of the participant characteristics and standard scores for each of the VABS-3 core domains. We then performed three separate multiple linear regressions to determine the contribution of the concurrent participant characteristics to the VABS-3 communication, daily living skills, and socialization standard scores. Effect sizes for predictors were described using Cohen’s *f*^2^ (0.02 = small effect, 0.15 = medium, 0.35 = large).

## Results

### Aim 1: patterns of adaptive skills in DS

Overall VABS-3 ABC and domain descriptive performance and range of scores are reported in Table 2. Qualitative descriptor classifications are provided in Table 3. There was a wide range of performance overall and on each domain that consisted of scores in the low, moderately low, and adequate adaptive skill classifications.

**Table 2:** Descriptive statistics for child characteristics (*n* = 44)

	M (SD)	Range
VABS-3 ABC SS	67.80 (9.37)	48–88
VABS-3 communication SS	60.16 (15.35)	32–87
VABS-3 daily living skills SS	67.34 (10.99)	33–95
VABS-3 socialization SS	77.43 (12.04)	46–104
DAS-II GCA	50.74 (11.24)	31–82
VABS-3 motor skills SS	68.55 (11.30)	33–87
SRS-2 total *T*-score	57.55 (8.88)	42–91

*VABS-3* Vineland Adaptive Behavior Scales, third edition, *ABC* Adaptive Behavior Composite, *SS* standard score, *DAS-II GCA* Differential Ability Scales, second edition General Conceptual Ability, *SRS-2* Social Responsiveness Scale, second edition

**Table 3:** Vineland Adaptive Behavior Scales-3 classifications of standard scores (*n* = 44)

	Classification		
	Low ***n*** (%)	Moderately low ***n*** (%)	Adequate ***n*** (%)
ABC	24 (54.5%)	19 (43.2)	1 (2.3%)
Communication	28 (63.6%)	15 (34.1%)	1 (2.3%)
Daily living skills	25 (56.9%)	17 (38.5%)	2 (4.6%)
Socialization	9 (20.5%)	25 (56.8%)	10 (22.7%)

*Note*. Vineland Adaptive Behavior Scales, third edition classifications and associated standard score ranges: low (20–70), moderately low (71–85), and adequate (86–114). *ABC*, Adaptive Behavior Composite

A repeated-measures ANOVA comparing mean standard score performance across the VABS-3 communication, daily living skills, and socialization domains demonstrated a significant effect of domain, *F*(2,86) = 44.93, *p* < 0.001, partial eta^2^ = 0.51. Follow-up analyses, using Bonferroni correction, indicated significant differences between all pairs of domain standard scores, with *p* < 0.01 for all domain comparisons. Specifically, the mean standard score for the socialization domain was significantly higher than the mean standard score for the daily living skills domain (*d* = 0.88), and the mean standard scores for both the socialization domain and the daily living skills domain were significantly higher than the mean standard score for the communication domain (*d* = 1.26 and *d* = 0.55 respectively) [socialization (S) > daily living skills (DL) > communication (C)].

**Fig. 1:**
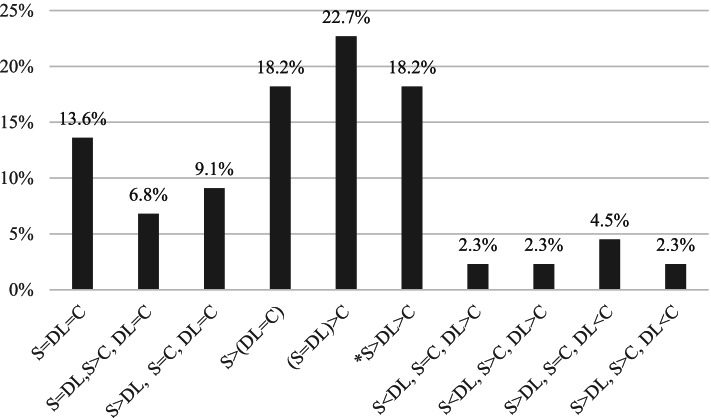
Frequency of individual adaptive skill patterns based on significant differences between Vineland Adaptive Behavior Scales, third edition domain standard scores. *C* communication domain, *DL* daily living skills domain, *S* socialization domain, “=”, difference not statistically significant, “<” or “>”, difference is statistically significant. *Group level-pattern: S > DL > C

Examination of individual participant performance was largely consistent with the group-level analyses. To determine the individual-level patterns of performance across domain scores for each child, VABS-3 Pairwise Difference Comparisons were computed. Ten individual-level patterns were identified. Although 52% of the sample demonstrated standard scores in the same rank order as in the group-level analyses (S > DL > C), these differences were statistically significant for only 18.2% of the participants. Two other partially overlapping patterns were about as common as the group-level pattern: one in which communication and daily living scores did not differ significantly from one another but were both significantly lower than the socialization score [S > (DL = C)], and one in which socialization and daily living scores did not differ significantly from one another but each was significantly higher than the communication score [(S = DL) > C]. These three patterns were all characterized by socialization scores that were significantly higher than communication scores and pattern differences were due to variability in daily living scores relative to the other domains. Across all participants, 70.5% demonstrated socialization scores significantly greater than communication scores (S > C), 52.3% demonstrated socialization scores significantly greater than daily living scores (S > DL), and 45.5% demonstrated daily living scores significantly greater than communication scores (DL > C). In Fig. 1, the specific patterns observed, with the corresponding proportions of occurrence, are described.

### Aim 2: child characteristics associated with adaptive skill

Descriptive data for participant characteristics are presented in Table 2. Bivariate associations between the putative predictor variables and adaptive skill are provided in Table 4. Putative predictors were entered simultaneously into regression models to further consider their unique contributions (Table 5).

**Table 4:** Correlations between standard scores for adaptive skills and other child characteristics (*n* = 42^a^)

	1	2	3	4	5	6
1. VABS-3 communication SS						
2. VABS-3 daily living skills SS	0.50**					
3. VABS-3 socialization SS	0.64**	0.58**				
4. Chronological age (years)	− 0.36*	0.14	− 0.29			
5. DAS-II GCA	0.60**	0.17	0.49**	− 0.51**		
6. VABS-3 motor skills SS	0.38*	0.65**	0.40**	0.12	0.06	
7. SRS-2 total *T*-score	− 0.61**	− 0.38*	− 0.62**	0.29	− 0.46**	− 0.25

*p* < 0.05*, *p* < 0.01**; ^a^VABS-3 Motor Skills data were not available for two participants

*VABS-3* Vineland Adaptive Behavior Scales, third edition, *DAS-II GCA* Differential Ability Scales, second edition General Conceptual Ability, *SS* standard score, *SRS-2* Social Responsiveness Scale, second edition

**Table 5:** Multiple regression models predicting adaptive skill standard scores (*n* = 42^a^)

	*B*	*SE B*	*p*	*β*	Semi-partial *r*	Cohen’s *f*^*2*^
**Communication** *(adjusted R*^2^ *= 0.53)*						
Chronological age	− 0.87	1.31	0.51	− 0.09	− 0.07	<0.01
DAS-II GCA	0.51	0.19	0.01*	0.38	0.30	0.04
VABS-3 motor skills SS	0.35	0.15	0.03*	0.27	0.25	0.02
SRS-2 total *T*-score	− 0.71	0.26	0.009**	− 0.36	− 0.31	0.05
**Daily living skills** *(adjusted R*^2^ *= 0.45)*						
Chronological age	1.75	1.06	0.11	0.23	0.20	<0.01
DAS-II GCA	.14	0.15	.36	0.14	0.11	<0.01
VABS-3 motor skills SS	0.54	0.12	<0.001**	0.54	0.51	0.28
SRS-2 total *T*-score	− 0.38	0.21	0.08	− 0.26	− 0.22	<0.01
**Socialization** *(adjusted R*^2^ *= 0.45)*						
Chronological age	− 0.64	1.15	0.58	− 0.08	− 0.07	<0.01
DAS-II GCA	0.27	0.17	0.11	0.24	0.19	<0.01
VABS-3 motor skills SS	0.31	0.14	0.03*	0.29	0.27	0.02
SRS-2 total *T*-score	− 0.67	0.23	0.005**	− 0.41	− 0.35	0.06

*p* < 0.05*, *p* < 0.01**; ^a^VABS-3 Motor Skills data were not available for two participants

*DAS-II GCA* Differential Ability Scales, second edition General Conceptual Ability, *VABS-3* Vineland Adaptive Behavior Scales, third edition, *SS* standard score, *SRS-2* Social Responsiveness Scale, second edition

The VABS-3 communication domain standard score regression model was significant, *F*(4, 39) =11.84, *p* < 0.001, *R*^2^_adj_ = 0.53. Cognitive ability (*p* = 0.01), motor skills (*p* = 0.03), and ASD symptomatology (*p* = 0.009) all significantly contributed to this model. The VABS-3 daily living skills domain regression model also was significant, *F*(4, 39) = 8.88, *p* < 0.001, *R*^2^_adj_ = 0.45. For this model, the only significant independent contributor was motor skills (*p* < 0.001). Finally, the regression model for the socialization domain was significant, *F*(4, 39) = 9.02, *p* < 0.001, *R*^2^_adj_ = 0.45. Both motor skills (*p* = 0.03) and ASD symptomatology (*p* = 0.005) uniquely accounted for variance in socialization performance. The effect size for each predictor is indicated in Table 5 and ranged from < 0.01 to 0.28.

## Discussion

The current study used the VABS-3 to examine the performance of young children with DS across adaptive skill domains at both the group level and the individual level and investigated child characteristics hypothesized to be sources of individual differences in adaptive skills. These data provide insight into the extent to which patterns of relative strengths and weaknesses are observed, allow for the identification of subgroups of children who may be overlooked when considering only group-level performance, and provide insight into other developmental domains that may be associated with adaptive skill performance.

### Patterns of adaptive skills in DS

When considering whether standard scores differed across adaptive skill domains, we found that at the group level, significant differences were observed among all pairs of domain scores, with socialization scores emerging as the highest standard score, followed by daily living, and then communication skills (S > DL > C). Considering the data at the individual level, we found that although approximately half of our sample demonstrated standard scores that were in the same rank order as the adaptive skill pattern identified by the group-level analyses, only 18% of our sample demonstrated a pattern in which the differences among the domains were statistically different (i.e., S > DL > C). Moreover, this pattern identified by the group-level analyses was one of 10 specific patterns observed at the individual level. Nonetheless, socialization skills emerged as an area of relative strength for most of the participants. Indeed, although standard scores ranged from 46 to 106, only 20.5% of children earned a socialization standard score classified as “low| (i.e., ≤ 70). Furthermore, 84.1% of the overall sample had significantly higher socialization scores compared to at least one other adaptive skill domain. These findings indicating a relative strength in socialization skills at both the group and individual levels replicate group-level findings from studies of adaptive skills in toddlers and young children with DS that used age-equivalent scores [16, 19, 28]. Importantly, this study provides a valuable contribution to the literature by reproducing the pattern of socialization strengths in young children with DS using standard scores, which do not suffer from the psychometric limitations associated with age-equivalent scores.

More variable findings emerged when considering patterns of daily living skill performance relative to the other adaptive skill domains. At the group level, the mean daily living skills standard score was significantly lower than the mean socialization standard score and significantly higher than the mean communication standard score. Standard scores ranged from 33 to 95, corresponding to classifications ranging from low to adequate, with most individuals (56.9%) earning standard scores in the ‘low’ category. At the individual level, a dominant pattern regarding where daily living skill performance scores fell compared to the other domains did not emerge. This individual-level variability may explain why less consistent findings have been observed in the relation of daily living skills to either socialization or communication skills within the literature [16, 19, 28]. For example, consider the individual patterns in performance observed in the current study. As shown in Fig. 1, 47.7% of our sample earned daily living scores that did not statistically differ from communication scores and 45.0% earned daily living scores that were significantly higher than communication scores. Similar patterns are observed in relation to socialization scores, with 43.1% of the sample earning daily living scores that did not statistically differ from socialization scores and 52.3% earning daily living scores that were significantly lower than socialization scores. Given these findings, it is not surprising that most of the other studies conducted to date have reported the same rank order pattern of performance, with variation with regard to whether or not significant differences were observed [16, 19]. There is only one study to date that has described daily living as the most significant area of challenge in children with DS, with performance significantly lower than both socialization and communication [28]; this pattern of performance was only observed in 2.3% of our sample.

Finally, communication was the lowest mean adaptive skill domain score; with standard scores ranging from 32 to 87, and 63.6% of participants in the “low” standard score category. Relative challenges for the communication domain have been previously described [16]; however, this is the first study to report this pattern of relative challenge using standard scores in children with DS. Moreover, we found that communication standard scores were significantly lower than standard scores for at least one other adaptive domain for almost three-quarters of our sample (72.8%). Nevertheless, the variation in individual strengths and challenges in children with DS highlights the substantial heterogeneity in this population and underscores the need to avoid overgeneralization of clinical expectations of adaptive skill for this population.

### Child characteristics associated with adaptive skill

We found similarities and differences across adaptive skill domains regarding the child characteristics that were unique predictors of individual differences. More specifically, motor skills emerged as a significant unique predictor of performance in all three core domains of adaptive skill. The effect size of motor skills in the daily living model was medium and considerably greater than any other effect sizes observed. A simple interpretation of this relation between motor and adaptive skills is that motor skills provide the necessary foundation for young children with DS to carry out communicative, daily living, and social tasks. Although this interpretation is theoretically aligned with the study (i.e., that foundational skills such as motor skills predict more complex adaptive skills), the study’s cross-sectional nature also allows for the inverse explanation that stronger adaptive skills promote motor skill development. It is also possible to consider that these systems are bidirectional, such that better motor skills lead to better adaptive skills, which in turn lead to further improved motor skills. This explanation is in line with Dynamic Systems Theory, which highlights the complex emergence of motor skills through interactions of factors within person and environment [37, 40]. Additional investigation of the specific motor skills that support adaptive skills and the associations between the sequences of milestones is warranted. These data may provide insight into potential intervention targets which, in turn, may promote independence for children with DS.

ASD symptomatology was also a significant unique predictor of both communication and socialization skills. Interestingly, the effects of ASD symptomatology scores remained even with the inclusion of other child characteristics known to be associated with increased ASD symptom risk, such as cognitive ability [6]. In our sample, ASD symptomatology was only a unique contributor to performance in the adaptive skill domains related to social interaction (i.e., socialization and communication skills). However, the ASD symptomatology predictor approached significance (*p* = 0.08) in relation to daily living skills, and with a larger sample size, it may have reached significance given the moderate correlation between the two variables (*r* = − 0.37). Although the present study was theoretically designed to suggest that ASD symptoms predict broader developmental skills (e.g., adaptive communication or socialization), we acknowledge that our data cannot determine the directionality of these relations. That is, although these data suggest that the increased presence of ASD symptomatology predicts challenges in adaptive communication and socialization, it is also possible that the opposite is true and limitations in adaptive communication and socialization skills impact the severity of ASD symptoms, such that children with low adaptive communication and socialization are more likely to have challenges related to ASD symptoms. Regardless of directionality, the relation between ASD symptomatology and adaptive skills highlights the need to increase the early detection of ASD symptoms and integrate interventions focused on social interaction for this population. This is especially of interest in a population like DS that is associated with an increased risk for ASD and ASD-related characteristics [5, 13]. If ASD symptoms are identified using symptom screeners, there is greater potential to intervene and support other related skills, such as adaptive socialization and communication. Furthermore, elucidating the association among these variables may be beneficial for developing treatments to support specific social and communicative adaptive skills in children with DS.

It was hypothesized that cognitive level would be associated with all adaptive skill domains, but surprisingly, cognitive level was only a significant unique predictor when considering adaptive communication skills. First, because language skills contribute to overall cognitive performance, it is no surprise that cognitive level would emerge as a unique contributor to adaptive communication skills. Moreover, it is well established that the development of nonverbal cognitive ability and communication skills are intricately intertwined. Not only does nonverbal cognitive ability support language development, but also language facilitates nonverbal cognitive development by establishing, organizing, and refining categories to access and use information from others [14, 44, 45]. Cognitive ability was also significantly correlated with communication and socialization, as expected [39], but the relation with daily living skills was not statistically significant. The lack of statistically significant association between cognitive level and daily living skills is an important finding from the investigation into child characteristics associated with adaptive domains. It may be that daily living skills at the developmental level of this sample do not require the same degree of cognitive abilities as do other adaptive domains. Continuing to expand on the specific child characteristics associated with adaptive performance and the specific contribution of cognitive level to adaptive skills will be an important direction for future research.

Finally, age was not a significant unique predictor of adaptive skills when utilizing standard scores. Even when considering bivariate correlations, the only significant association observed was a negative association between age and communication skills. It is important to recognize that standard scores are not inherently expected to change with age. In particular, if an individual continues to develop at the same rate relative to his or her peers (i.e., those represented in the norming sample), then an IQ score would be expected to stay stable. In contrast, when there is a change in the rate that an individual is developing relative to his or her peers, that individual’s IQ score would change. In the present study, the negative association between age and communication skills observed in children with DS suggests that the rate at which communication skills are developing decreases over time relative to the rate expected by the general-population norms. This finding is consistent with prior literature regarding the language and communication delays observed in children with DS [8]. However, findings from the regression model also indicate that this association did not account for enough of the overall variance in communication skills to emerge as a unique contributor when simultaneously considering the contributions of overall cognitive performance, motor skills, and ASD symptom severity. It remains possible that at different developmental periods, and/or across a wider age range, the influence of age on standard score performance would make a significant unique contribution to adaptive communication standard score. Future research considering longitudinal changes in adaptive skill standard scores and age-related changes in the associations between child characteristics (i.e., cognitive ability, motor skills, and ASD symptomatology) are needed to support the cross-sectional findings from the present study.

## Limitations and future directions

Although this study is the first to describe heterogeneity and sources of variability in adaptive skill performance in young children with DS, there are multiple study limitations. First, cross-sectional data were used, limiting the analyses to associations among variables, and the directionality of relations could not be investigated. The present study examined VABS-3 domain scores; however, analyses of subscale scores were not conducted given the large number of tests that would be required relative to the sample size and differences in the range of v-scale scores possible for each subscale across the ages studied. Future studies with a larger sample size across a somewhat older age span should investigate patterns of performance on the VABS-3 subscales to provide additional precision in characterizing adaptive skills to further inform intervention for children with DS. Additionally, there was a slight overlap in the ASD symptomatology measure (SRS-2) and VABS-3 Socialization domain at the item level. For example, both measures queried the child’s social interactions and dependence on adults. This overlap was minimal and not determined to compromise the study’s analytic plan. It is important to note that these two instruments (VABS-3 and SRS-2) are parent-report measures; adding direct assessment of these domains would provide a broader viewpoint that may highlight different subtleties in child adaptive or social behavior. Participants also had predominantly upper middle or high SES, with minimal representation from low SES (only 6.9% below $50,000 USD annual family income). Race and ethnicity were primarily White and non-Hispanic, yet there was representation from other groups, especially White or other and Hispanic (20.4%). Inclusion criteria for the broader study may have limited participation because basic motor competencies were required to complete study procedures. Thus, we may not have the full range of performance included. However, participants in the sample did have a wide range of motor ability. This limitation pertains to all child characteristics, as the variability in cognitive ability, motor skills, or ASD symptomatology may have impacted the group-level and individual-level patterns of adaptive skills observed. Future studies should evaluate adaptive skills longitudinally and determine if (and if so, how) patterns of relative strength and weakness shift throughout development and if age-related changes are observed in the amount of variability in individual performance. Latent Profile Analysis will be an important tool for future studies to examine within-group patterns of adaptive skills further and establish child characteristics associated with adaptive skill profiles.

## Conclusions

This study makes key contributions to the characterization of adaptive skills in DS at both the group and individual levels. Across the adaptive skill domains, socialization was maintained as a strong adaptive skill relative to communication and daily living. While this group-level pattern also was one of the most common patterns at the individual level, two slightly different patterns were equally prevalent at the individual level as well as several less frequent patterns, emphasizing the heterogeneity observed in this population and need for interventions to be individualized based on the child’s relative strengths and weaknesses. Child characteristics were also considered in relation to adaptive skills, and motor skills were the most salient predictor of adaptive skills across domains. ASD symptoms also contributed significant unique variance to adaptive communication and socialization. These findings highlight the different developmental mechanisms that may support adaptive skills, but further investigation is required to determine the directionality of these relations. In anticipation of challenges with adaptive skills in DS, motor and social skills should be considered as targets for intervention to promote independence in this population.

## Data Availability

The datasets used and analyzed during the current study are available from the corresponding author on reasonable request.

## Abbreviations

DS: Down syndrome
ASD: Autism spectrum disorder
VABS-3: Vineland Adaptive Behavior Scales, third edition
C: VABS-3 communication domain
DL: VABS-3 daily living skills domain
S: VABS-3 socialization domain
ABC: Adaptive Behavior Composite
SS: Standard score
DAS-II: Differential Ability Scales, second edition
GCA: General Conceptual Ability
SRS-2: Social Responsiveness Scale, second edition
